# Comparison of Meibomian Gland Loss Between Pediatric Patients with Blepharo-Keratoconjunctivitis and Age-Matched Controls Using Non-Contact Meibography

**DOI:** 10.3390/jcm15176891

**Published:** 2026-09-06

**Authors:** Jaeuk Jeong, Ji Sun Paik, Kyung Sun Na, Kyongjin Cho

**Affiliations:** 1 Department of Ophthalmology, Dankook University Hospital, Dankook University College of Medicine, Cheonan 31116, Republic of Korea; jaeuk@dkuh.co.kr; 2 Department of Ophthalmology, Yeouido St. Mary’s Hospital, College of Medicine, The Catholic University of Korea, Seoul 06591, Republic of Korea

**Keywords:** blepharo-keratoconjunctivitis, meibomian gland dysfunction, meibography, allergic conjunctivitis, pediatric ocular surface disease

## Abstract

**Objectives:** To compare the extent of meibomian gland loss in pediatric patients with blepharo-keratoconjunctivitis (BKC), allergic conjunctivitis, and healthy controls using non-contact infrared meibography. **Methods:** This retrospective observational study included 81 patients aged 18 years or younger: 20 with BKC, 29 with allergic conjunctivitis, and 32 healthy controls. Meibomian gland loss in the upper and lower eyelids was quantified on non-contact infrared meibography images. Right and left eyes were analyzed separately, and only eyes with the same laterality were compared among groups. **Results:** Meibomian gland loss was significantly greater in the BKC group than in the allergic conjunctivitis and control groups in all four eyelids (right, left, upper and lower; all *p* < 0.001). In contrast, gland loss in allergic conjunctivitis showed intermediate values between BKC and controls and did not differ significantly from controls in some eyelids. Within the BKC group, meibomian gland loss did not differ significantly between eyes with and without overt corneal or conjunctival lesions (*p* ≥ 0.05). **Conclusions:** Pediatric patients with BKC demonstrate marked meibomian gland loss compared with allergic conjunctivitis and healthy eyes. These findings suggest that BKC represents a particularly aggressive form of MGD. Importantly, early recognition and appropriate treatment of BKC in pediatric patients may help prevent recurrent inflammation and halt further meibomian gland loss, thereby reducing long-term MGD.

## 1. Introduction

Blepharo-keratoconjunctivitis (BKC) is a chronic, recurrent inflammatory disorder of the ocular surface characterized by blepharitis, meibomian gland dysfunction (MGD), and conjunctival and corneal inflammation, predominantly affecting children and young adults [1]. This disease originates from the eyelid margin and progressively extends to the conjunctiva and cornea, where it can result in keratoconjunctivitis, corneal scarring and neovascularization [2,3]. Without timely diagnosis and appropriate management, BKC may progress to intractable corneal opacities, astigmatism, and amblyopia, leading to substantial visual morbidity [4].

MGD is a major contributor to dry eye disease, as instability of the meibum lipid layer accelerates tear film evaporation and exacerbates ocular surface inflammation [5,6]. Recent clinical practice guidelines highlight MGD as a common, clinically important disorder that substantially impairs quality of life and requires systematic diagnostic and therapeutic approaches [7]. The introduction of non-contact infrared meibography has enabled quantitative assessment of meibomian gland morphology, and several studies have demonstrated that meibomian gland loss correlates significantly with MGD severity and ocular surface disease [7,8,9,10]. Using meibography, previous investigations have shown that young patients with BKC exhibit more pronounced meibomian gland loss and structural alterations compared with healthy controls and demonstrate more severe MGD characteristics than those with other forms of blepharitis [11]. These findings suggest that BKC should not be regarded merely as simple blepharitis but rather as a destructive subtype of MGD that causes irreversible structural damage to the meibomian glands [12].

Allergic conjunctivitis is another common chronic inflammatory ocular surface disease in children. Accumulating evidence indicates that prolonged allergic inflammation can adversely affect meibomian gland morphology and function [13,14]. In pediatric patients with allergic conjunctivitis, the prevalence of severe meibomian gland atrophy is significantly higher than in healthy controls (51.67% vs. 10%), and the severity of atrophy increases with longer disease duration [13]. Adult patients with allergic conjunctivitis also demonstrate significantly increased meibomian gland duct distortion and morphological alterations compared with controls [14]. However, most previous studies have focused on single disease entities or comparisons between different MGD phenotypes, and direct comparative data on meibomian gland loss among young patients with BKC, allergic conjunctivitis, and healthy controls remain limited [15].

This study investigated meibomian gland loss and morphological changes in pediatric patients with BKC, allergic conjunctivitis, and healthy controls using non-contact infrared meibography to determine the extent of meibomian gland loss across these groups and to broaden the understanding of structural damage associated with BKC, which may aid in early diagnosis and follow-up of these patients.

## 2. Materials and Methods

This retrospective, observational study adhered to the tenets of the Declaration of Helsinki and was approved by the Institutional Review Board (IRB) of Dankook University Hospital (IRB approval number: 2026-01-023-006) and Yeouido St. Mary’s Hospital (IRB approval number: SC26RIDI0088). The requirement for written informed consent was waived by the IRB because the study involved retrospective analysis of de-identified medical records without additional patient intervention.

### 2.1. Study Population

This study included patients aged 18 years or younger who were diagnosed with BKC or allergic conjunctivitis by ophthalmologists at Dankook University Hospital and Yeouido St. Mary’s Hospital between January 2020 and January 2026 and who underwent non-contact infrared meibography as part of their clinical evaluation. In some cases of BKC, phlyctenulosis was observed as an associated finding. Age-matched healthy controls without clinical evidence of ocular surface disease who underwent meibography during the same period were also included. In total, 81 participants were enrolled: 20 with BKC, 29 with allergic conjunctivitis, and 32 healthy controls.

### 2.2. Inclusion Criteria

#### 2.2.1. BKC Group

**Figure 1:**
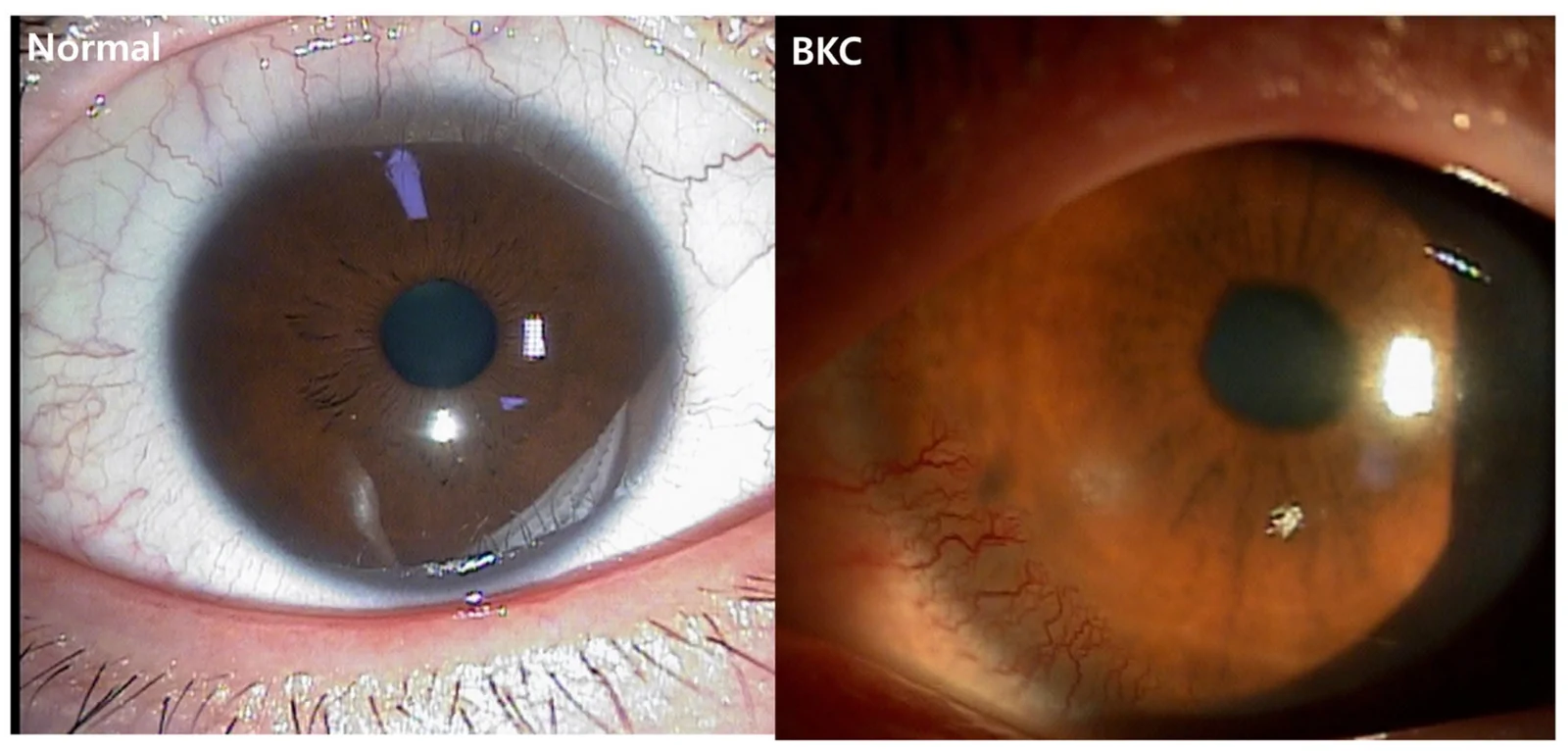
Slit-lamp photographs of healthy control and patient with BKC, showing corneal opacity and neovascularization in patient with BKC.

**Figure 2:**
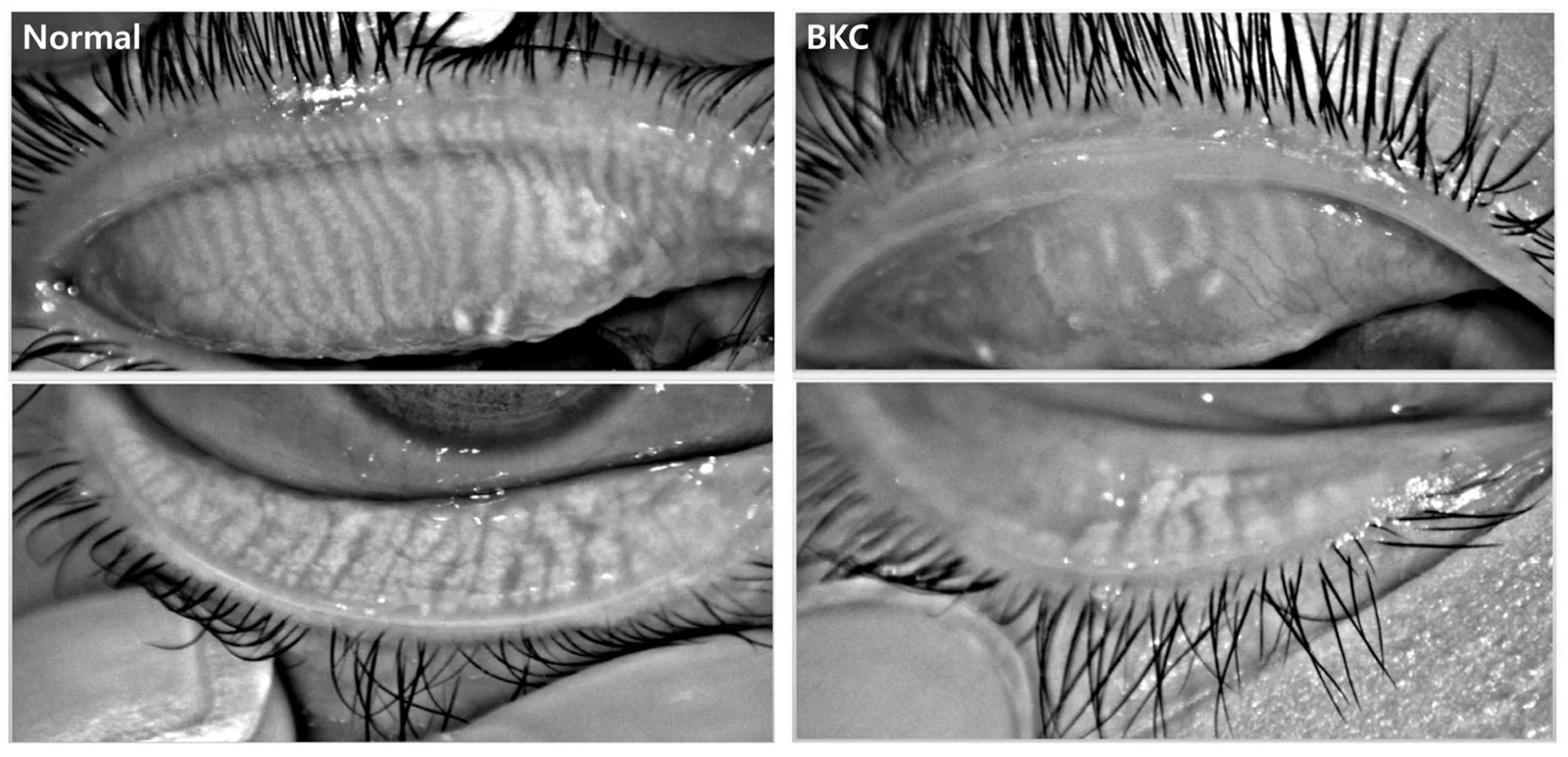
Non-contact infrared meibography images of the upper and lower eyelids in healthy control and patient with BKC, demonstrating marked meibomian gland loss and distortion in patient with BKC.

Patients with a clinical diagnosis of BKC based on slit-lamp biomicroscopy findings consistent with chronic blepharitis and MGD accompanied by conjunctival and/or corneal inflammation, such as conjunctival hyperemia, superficial punctate keratitis, phlyctenules, or peripheral corneal infiltrates (Figure 1 and Figure 2) [1,2,3].

#### 2.2.2. Allergic Conjunctivitis Group

Patients with a clinical diagnosis of allergic conjunctivitis, including seasonal allergic conjunctivitis, perennial allergic conjunctivitis, atopic keratoconjunctivitis, or vernal keratoconjunctivitis, based on a history of allergic symptoms of itching and tearing and characteristic conjunctival signs such as papillae [13,14].

#### 2.2.3. Control Group

Age-matched subjects without a history of ocular surface disease, blepharitis, or allergic eye disease, and with normal findings on slit-lamp examination, who underwent non-contact infrared meibography during routine ophthalmic evaluation.

### 2.3. Exclusion Criteria

Patients were excluded if they met any of the following criteria:

(1) presence of other ocular surface diseases known to affect meibomian gland morphology, such as Stevens–Johnson syndrome, ocular cicatricial pemphigoid, or trachoma [5,10,16]; (2) history of eyelid anomalies, eyelid surgery, eyelid trauma, or chemical injury [16]; (3) chronic systemic conditions associated with MGD, including juvenile rosacea or systemic autoimmune diseases [16]; (4) inadequate quality of meibography images precluding reliable quantitative analysis [8,9,11].

Dry eye disease was not independently excluded from the BKC and allergic conjunctivitis groups. In contrast, controls with a documented diagnosis of dry eye disease or dry-eye-related ocular surface abnormalities on slit-lamp examination were excluded based on medical-record review.

### 2.4. Clinical Examination and Data Collection

Clinical data, including age, sex, and corrected visual acuity (CV), were collected from the medical records. Meibomian gland loss measurements derived from meibography images were compared among the three diagnostic groups.

CV was measured in decimal notation during routine clinical examination.

Meibography images were obtained using the OCULUS Keratograph^®^ 5M (OCULUS Optikgeräte GmbH, Wetzlar, Germany) and LipiView^®^ II Ocular Surface Interferometer (Johnson & Johnson Vision, Santa Ana, CA, USA). The images were then analyzed by ImageJ software (version 1.53, National Institutes of Health, Bethesda, MD, USA). For each eyelid, the expected full area of each meibomian gland was estimated with reference to intact glands in the same eyelid [8]. When intact glands were insufficient for reference, the presumed contour was estimated according to the anatomical arrangement and orientation of the meibomian glands within the eyelid. Meibography measurements were performed by two ophthalmologists who were not masked to the clinical diagnosis or group assignment. Each ophthalmologist initially evaluated the images independently, and the final measurements used for analysis were determined by consensus. Formal inter-observer and intra-observer reproducibility assessments were not performed. The extent of meibomian gland preservation was quantified as the percentage ratio of the total residual gland area to the estimated full meibomian gland area within the eyelid.

### 2.5. Statistical Analysis

To avoid issues related to inter-eye correlation, right eyes and left eyes were analyzed separately, and only eyes within the same laterality (right–right and left–left) were compared across the three groups. Upper and lower eyelid meibography images were analyzed separately.

Statistical analyses were performed using Python (version 3.14.2; Python Software Foundation, Wilmington, DE, USA). Continuous variables that did not follow a normal distribution were summarized as medians and interquartile ranges and compared among the three groups using the Kruskal–Wallis test, followed by post hoc pairwise comparisons using the Mann–Whitney U test with Bonferroni correction. Categorical variables were presented as frequencies and percentages and compared using the chi-square test, followed by pairwise comparisons using chi-square tests with Bonferroni correction. Within the BKC group, we further compared meibomian gland loss between eyes with and without ocular surface lesions, defined as the presence of corneal and/or conjunctival staining, using the independent *t*-test. Normality of continuous variables for the independent *t*-test comparison within the BKC group was assessed using the Shapiro–Wilk test.

To evaluate the independent association between diagnostic group and meibomian gland loss, multivariable linear regression analyses were performed separately for the right upper lid (RUL), right lower lid (RLL), left upper lid (LUL), and left lower lid (LLL). Meibomian gland loss was modeled as the dependent variable, with diagnostic group, age, and sex included as independent variables. The control group and male sex were used as the reference categories. A *p*-value < 0.05 was considered statistically significant for all analyses.

## 3. Results

A total of 81 patients were enrolled in this study, including 20 with BKC, 29 with allergic conjunctivitis, and 32 healthy controls. Patients with unreliable meibography images, such as those with large papillae or other findings that obscured accurate visualization of the meibomian glands, were excluded from the analysis.

Baseline characteristics of the three groups are summarized in Table 1. Age differed significantly among the three groups (*p* = 0.0165). Post hoc pairwise comparisons with Mann–Whitney U test with Bonferroni correction showed that the allergic conjunctivitis group was significantly younger than the BKC group (adjusted *p* = 0.0205), whereas no significant age differences were observed between the BKC and control groups (adjusted *p* = 1.000) or between the allergic conjunctivitis and control groups (adjusted *p* = 0.0841). The proportion of female patients was higher in the BKC group than in the allergic conjunctivitis and control groups, but the overall comparison of sex distribution among the three diagnostic groups did not reach statistical significance (*p* = 0.057). However, when the allergic conjunctivitis and control groups were combined into a single “others” category, a separate chi-square analysis comparing the BKC group with the other groups demonstrated a statistically significant difference in sex distribution (*p* = 0.037). Given this imbalance in age and sex distribution, we performed additional multivariable analysis to assess whether age and sex confounded the relationship between diagnostic group and meibomian gland loss.

**Figure 3:**
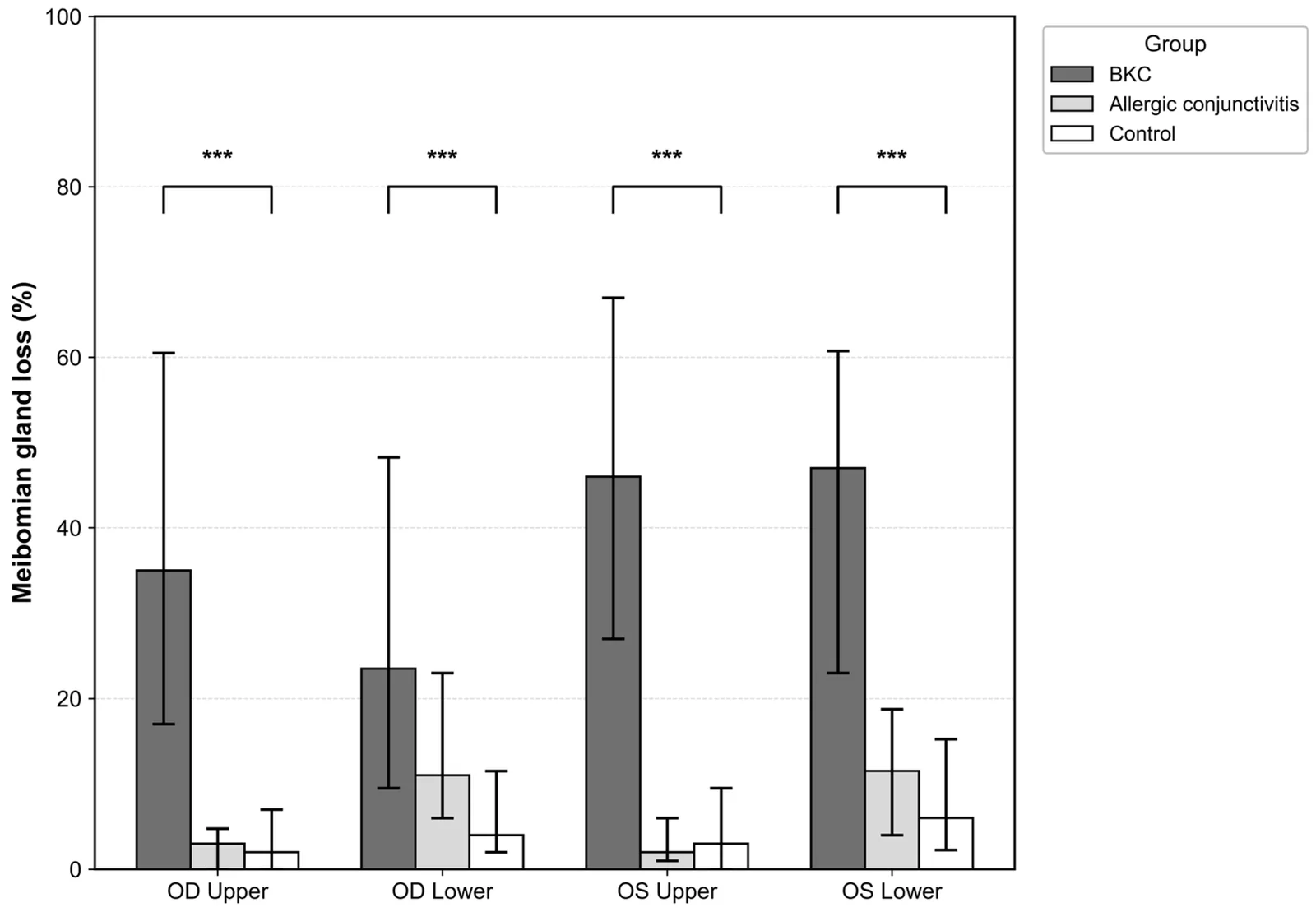
Meibomian gland loss in the upper and lower eyelids of the right and left eyes in patients with BKC, allergic conjunctivitis, and healthy controls. Bars represent median percentages of meibomian gland loss with interquartile ranges for each group in the right upper lid (OD_Upper), right lower lid (OD_Lower), left upper lid (OS_Upper), and left lower lid (OS_Lower). Significant differences among groups are indicated by asterisks (***, *p* < 0.001).

The percentages of meibomian gland loss in each eyelid are presented in Table 2 and Figure 3. Meibomian gland loss differed significantly among the BKC, allergic conjunctivitis, and control groups in all four eyelids (all Kruskal–Wallis *p* < 0.001). In Bonferroni-adjusted post hoc pairwise comparisons, meibomian gland loss was significantly greater in the BKC group than in the control group in all four eyelids (all adjusted *p* < 0.001). Compared with the allergic conjunctivitis group, the BKC group showed significantly greater meibomian gland loss in the RUL, LUL, and LLL (all adjusted *p* < 0.001), whereas the difference in the RLL did not remain significant after Bonferroni correction (adjusted *p* = 0.059). Meibomian gland loss did not differ significantly between the allergic conjunctivitis and control groups in any eyelid (all adjusted *p* > 0.05; Supplementary Table S1). In multivariable linear regression models adjusted for age and sex, BKC was significantly associated with greater meibomian gland loss than the control group in all four eyelids (all *p* < 0.001). Detailed regression coefficients, standard errors, 95% confidence intervals and model-fit statistics are presented in Supplementary Table S2.

**Figure 4:**
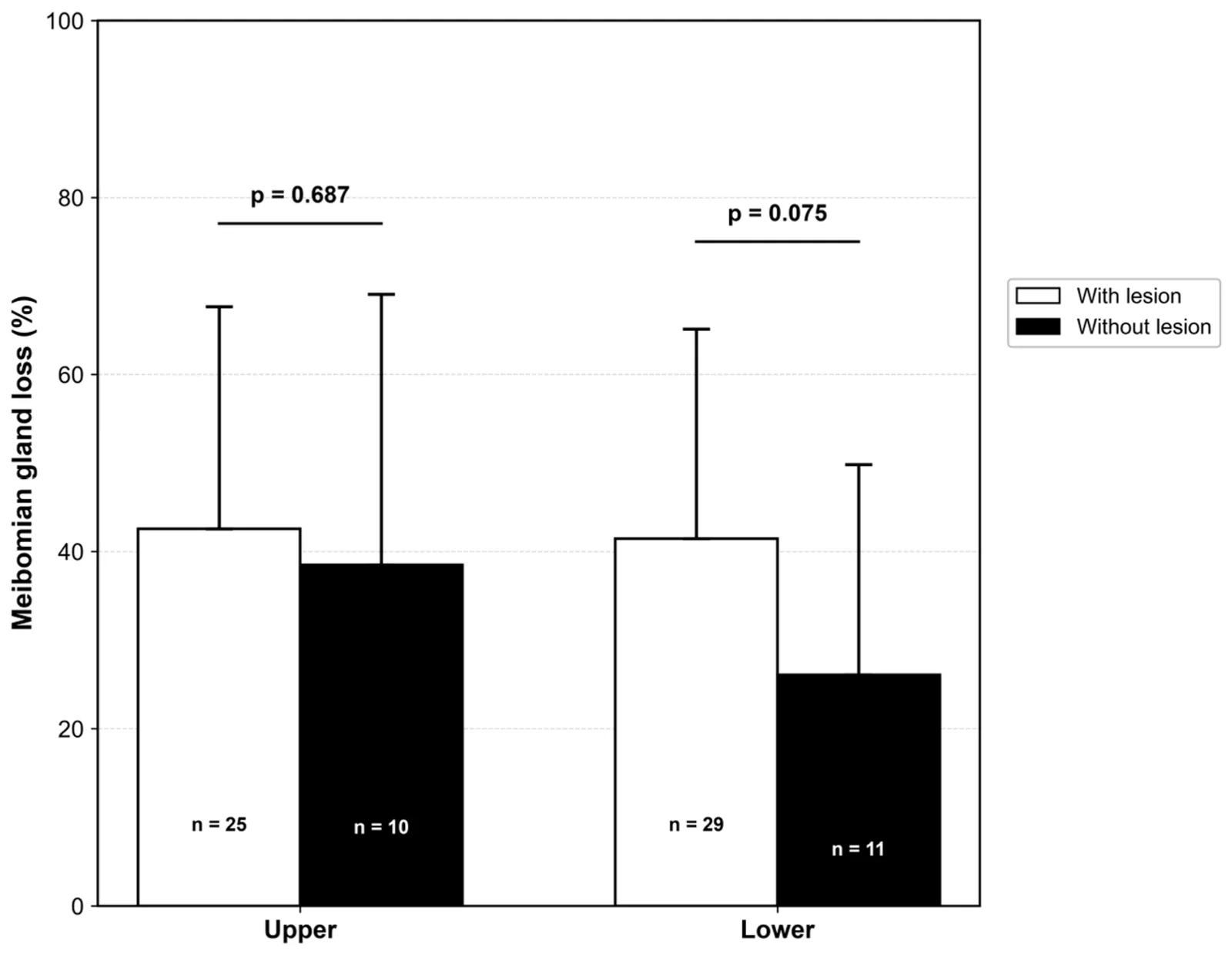
Comparison of meibomian gland loss between eyes with and without corneal or conjunctival lesions in patients with BKC. Bars represent mean percentages of meibomian gland loss with standard deviations in the upper and lower eyelids of eyes with lesions (white bars) and without lesions (black bars). The number of eyes in each subgroup (*n*) is shown within the bars, and *p*-values indicate the results of between-group comparisons for upper and lower lids, respectively.

Within the BKC group, meibomian gland loss was further compared between eyes with and without corneal or conjunctival lesions. In eyes with lesions, mean meibomian gland loss was 42.56 ± 25.11% in the upper lids and 41.45 ± 23.70% in the lower lids, whereas in eyes without lesions, it was 38.50 ± 30.57% in the upper lids and 26.09 ± 23.73% in the lower lids. These differences were not statistically significant for either the upper lids (*p* = 0.700) or the lower lids (*p* = 0.083) (Table 3, Figure 4).

## 4. Discussion

In this study, we found that pediatric patients with BKC demonstrated significantly greater meibomian gland loss compared to those with allergic conjunctivitis and healthy controls, indicating that BKC represents a particularly destructive form of ocular surface disease affecting the meibomian glands. Although the BKC group had a higher proportion of female patients, multivariable analyses adjusting for sex confirmed that the association between BKC and increased meibomian gland loss remained significant, while sex itself was not a significant predictor. This suggests that the observed meibomian gland loss is primarily related to disease status rather than sex distribution.

This pattern of findings provides additional insight into the potential nature of meibomian gland involvement in pediatric BKC. The greater meibomian gland loss observed in the BKC group across the eyelids, even after adjustment for sex and age, indicates that BKC was associated with more extensive meibomian gland loss in this study. Although the present cross-sectional data cannot establish the temporal sequence or causal mechanisms underlying this association, the findings are compatible with the possibility that chronic lid margin inflammation, meibomian gland obstruction, and recurrent inflammatory episodes in BKC contribute to cumulative glandular damage [12]. In contrast, the absence of a statistically significant difference between the allergic conjunctivitis and healthy control groups should not be interpreted as evidence that allergic conjunctivitis has no effect on the meibomian glands. Rather, meibomian gland involvement in allergic conjunctivitis may be heterogeneous and influenced by factors not assessed in the present analysis, including disease subtype, inflammatory activity, duration of disease, eye-rubbing behavior, and prior treatment [13]. Taken together, our findings indicate that, within this cohort, BKC is associated with the most pronounced meibomian gland loss among the evaluated pediatric ocular surface conditions.

BKC is a chronic, recurrent inflammatory disorder that originates from the eyelid margin and progresses to involve the conjunctiva and cornea [2]. The initial presentation typically includes anterior and posterior blepharitis along with recurrent chalazion, reflecting the chronic inflammatory nature of the disease [15]. The underlying pathophysiology is multifactorial, encompassing MGD, bacterial colonization, vascular changes, type IV hypersensitivity reactions, immune dysregulation, Demodex mite infestation, genetic predisposition, and environmental factors [1].

Among these pathogenic factors, MGD plays a particularly central role, leading to tear film lipid deficiency and inflammatory pathway activation [5,6]. Meibomian glands synthesize fatty alcohols, aldehydes and fatty acids that are key precursors of wax and cholesteryl esters, the major lipids of meibum, and disturbances in these pathways can destabilize the tear film and ocular surface [17]. In addition, ectodysplasin A–related signaling has been implicated in meibomian gland development and ocular surface homeostasis [18]. The development of MGD in BKC involves multiple mechanisms that result in structural and functional impairment, including bacterial toxin-mediated damage, ductal obstruction, inflammatory cell infiltration, and subsequent glandular atrophy [19,20,21]. Inflammatory mediators in MGD create a vicious cycle of inflammation and gland destruction that perpetuates BKC progression [22,23]. Previous studies have demonstrated that BKC patients exhibit more severe MGD characteristics compared to other forms of blepharitis, with irreversible structural loss being a defining feature, thereby emphasizing the importance of early diagnosis and treatment [3,12].

Generally, BKC is considered to be associated with more pronounced meibomian gland loss than other ocular surface diseases [2,12,23]. Yue et al. reported that patients with BKC had worse meibomian gland function and a higher prevalence of medical histories related to meibomian gland disease compared with healthy individuals, which is in line with the findings of the present study [12]. Allergic conjunctivitis has also been shown to induce a moderate degree of meibomian gland loss [13,14]. In a study by Wanfeng et al., meibomian glands in eyes with allergic conjunctivitis demonstrated significantly greater gland loss than in control eyes, whereas in our series the extent of gland loss did not differ significantly between allergic conjunctivitis and healthy controls, possibly reflecting differences in disease severity, duration, or age distribution among study populations [13,14]. To our knowledge, few studies have directly compared meibomian gland loss between clinically affected and unaffected fellow eyes in pediatric BKC. In the present series, meibomian gland loss did not differ significantly between eyes with and without overt corneal or conjunctival lesions. However, lower-lid gland loss was numerically greater in eyes with lesions than in those without lesions (41.45% vs. 26.09%; *p* = 0.083). Given the limited subgroup sample size, this finding should be interpreted cautiously, and larger studies are needed to clarify the relationship between ocular surface lesions and meibomian gland loss in pediatric BKC. Nevertheless, the absence of overt corneal or conjunctival lesions should not be assumed to indicate normal meibomian gland status. Careful evaluation of both eyes, including eyes without clinically apparent ocular surface lesions, may therefore be warranted in children with BKC, because the absence of clinically apparent inflammation in one eye does not necessarily indicate normal meibomian gland status. Assessment of meibomian gland status should therefore not rely solely on the laterality of corneal involvement [3,4,12]. Because of the cross-sectional design, the present study could not determine whether meibomian gland loss precedes, accompanies, or persists after clinically apparent ocular surface lesions. Longitudinal studies with standardized assessments of disease activity and treatment are needed to clarify these relationships [3,4,12].

In clinical practice, the present findings underscore the importance of early recognition of BKC in pediatric patients, as meibomian gland loss is generally considered irreversible once established [3]. Early diagnosis and prompt initiation of appropriate anti-inflammatory therapy and lid hygiene may help preserve meibomian gland structure and function, stabilize the tear film, and reduce the risk of long-term ocular surface damage [6]. Non-contact infrared meibography provides a non-invasive and relatively objective method for visualizing meibomian gland morphology and may assist in differentiating severely destructive phenotypes such as BKC from milder entities such as allergic conjunctivitis, as well as in monitoring structural progression over time [8,9,10,11]. The present results also support the need for an intensive and sustained treatment approach in pediatric BKC, with long-term follow-up to detect recurrent inflammation, progressive gland loss, and associated corneal changes at an early stage, which is essential for the prevention of amblyopia and other sight-threatening sequelae in this population [3].

The present study has several limitations. First, its retrospective design inherently carries a risk of selection bias, as only patients who underwent meibography as part of their routine clinical care were included. Second, meibography images were analyzed by manual delineation of residual gland areas and estimation of the expected full gland area. Although measurements were independently reviewed by two ophthalmologists and finalized by consensus, the graders were not masked to the clinical diagnosis or group assignment, and formal inter-observer and intra-observer reproducibility assessments were not performed. Therefore, observer-dependent measurement bias cannot be excluded. Third, the cross-sectional nature of the study does not allow assessment of longitudinal changes or progression of meibomian gland loss, and prospective follow-up studies are required to clarify the temporal course of gland damage in pediatric BKC and allergic conjunctivitis. Fourth, the relatively small size of each subgroup warrants cautious interpretation of the results and underscores the need for larger, preferably multicenter investigations. Fifth, contact lens use was not assessed separately and was not controlled for in this analysis, which may have introduced additional variability in ocular surface and meibomian gland findings. Finally, disease severity and duration were not systematically incorporated into the analysis. In addition, the allergic conjunctivitis group included several clinical subtypes, including seasonal and perennial allergic conjunctivitis, atopic keratoconjunctivitis, and vernal keratoconjunctivitis, which may differ in disease severity and their effects on meibomian gland morphology. Future studies integrating detailed grading of clinical severity, symptom burden, and disease duration may help to elucidate the relationship between meibomian gland morphology, ocular surface inflammation, and long-term visual outcomes.

## 5. Conclusions

Pediatric patients with BKC exhibited substantially greater meibomian gland loss than those with allergic conjunctivitis and healthy controls, confirming that BKC is associated with marked structural damage to the meibomian glands. Non-contact infrared meibography provided an objective, image-based method for visualizing and quantifying this structural loss and may serve as a useful tool for identifying severely affected patients and monitoring disease course. These findings emphasize the importance of early diagnosis and aggressive, sustained treatment in pediatric BKC in order to preserve meibomian gland structure and reduce the risk of long-term ocular surface complications.

## Figures and Tables

**Table 1:** Comparison of baseline characteristics between BKC, allergic conjunctivitis and control groups.

	BKC (*n* = 20)	Allergic Conjunctivitis (*n* = 29)	Control (*n* = 32)	*p*-Value
Age	13.0 (11.8–15.0)	10.0 (9.0–12.0)	12.0 (9.8–14.5)	0.0165 ^a^
Sex	3:17 (F: 85%)	12:17 (F: 58.6%)	15:17 (F: 53.1%)	0.0571 ^b^
CV-OD	0.90 (0.55–1.00)	0.90 (0.80–1.00)	1.00 (0.88–1.00)	0.443 ^a^
CV-OS	0.90 (0.60–0.90)	1.00 (0.90–1.00)	1.00 (0.88–1.20)	0.0155 ^a^

Data are presented as median and interquartile range for continuous variables and *n* (%) for categorical variables. BKC: blepharo-keratoconjunctivitis, CV: corrected vision, OD: oculus dexter, OS: oculus sinister. ^a^—Kruskal–Wallis test result. ^b^—Pearson chi-square test result. *n* = number of patients.

**Table 2:** Comparison of average meibomian gland loss between BKC, allergic conjunctivitis and control groups.

	BKC	Allergic Conjunctivitis	Control	*p*-Value
	(*n* = 20)	(*n* = 29)	(*n* = 32)	
MG loss (RUL, %)	35.00 (17.00–60.50)	3.00 (0.00–4.75)	2.00 (0.00–7.00)	0.000020 ^a^
	(*n* = 18)	(*n* = 26)	(*n* = 31)	
MG loss (RLL, %)	23.50 (9.50–48.25)	11.00 (6.00–23.00)	4.00 (2.00–11.50)	0.000373 ^a^
	(*n* = 20)	(*n* = 29)	(*n* = 32)	
MG loss (LUL, %)	46.00 (27.00–67.00)	2.00 (1.00–6.00)	3.00 (0.00–9.50)	0.000000 ^a^
	(*n* = 17)	(*n* = 28)	(*n* = 31)	
MG loss (LLL, %)	47.00 (23.00–60.75)	11.50 (4.00–18.75)	6.00 (2.25–15.25)	0.000001 ^a^
	(*n* = 20)	(*n* = 28)	(*n* = 30)	

Data are presented as median and interquartile range. BKC: blepharo-keratoconjunctivitis, RUL: right upper lid, RLL: right lower lid, LUL: left upper lid, LLL: left lower lid. ^a^—Kruskal–Wallis test result. *n* = number of patients.

**Table 3:** Comparison of average meibomian gland loss between with lesions and without lesions in the BKC group.

	With Lesions	Without Lesions	*p*-Value
Upper lid MG loss (%)	42.56 ± 25.11 (*n* = 25)	38.50 ± 30.57 (*n* = 10)	0.687 ^a^
Lower lid MG loss (%)	41.45 ± 23.70 (*n* = 29)	26.09 ± 23.73 (*n* = 11)	0.075 ^a^

Data are presented as respective percent as mean value ± standard deviation. MG: meibomian gland. ^a^—Independent *t*-test result. *n* = number of eyes.

## Data Availability

The data presented in this study are not publicly available because they contain potentially identifiable clinical information from participants. De-identified data may be available from the corresponding authors upon reasonable request and subject to Institutional Review Board approval.

## Supplementary Materials

The following supporting information can be downloaded at: https://www.mdpi.com/article/10.3390/jcm15176891/s1, Supplementary Table S1. Bonferroni-adjusted post hoc pairwise comparisons of meibomian gland loss among the BKC, allergic conjunctivitis, and control groups. Supplementary Table S2. Multivariable linear regression analyses of meibomian gland loss in each eyelid. Diagnostic group, age, and sex were included as covariates. The control group and male sex were used as the reference categories. Values are adjusted β coefficients (standard errors), followed by 95% confidence intervals in parentheses: β (SE), (lower 95% CI, upper 95% CI).
