# Supplementary material for: Comparison of Meibomian Gland Loss Between Pediatric Patients with Blepharo-Keratoconjunctivitis and Age-Matched Controls Using Non-Contact Meibography
Source: J Clin Med. 2026 Sep 6;15(17):6891. doi: 10.3390/jcm15176891 (PMC13566522; doi:10.3390/jcm15176891)
Supplement: Supplementary file 1 [file jcm-15-06891-s001.zip › Supplementary Table S1_Table2 post hoc.pdf]

	Comparision	n (first group)	n (second group)	Mann-Whitney U	Adjusted p-value
RUL	BKC vs allergic conjunctivitis	18	26	398.0	<0.001
	BKC vs control	18	31	485.5	<0.001
	Allergic conjunctivitis vs control	26	31	415.0	1.000
RLL	BKC vs allergic conjunctivitis	20	29	405.0	0.059
	BKC vs control	20	32	520.5	<0.001
	Allergic conjunctivitis vs control	29	32	607.5	0.115
LUL	BKC vs allergic conjunctivitis	17	28	443.5	<0.001
	BKC vs control	17	31	500.5	<0.001
	Allergic conjunctivitis vs control	28	31	417.5	1.000
LLL	BKC vs allergic conjunctivitis	20	28	473.0	<0.001
	BKC vs control	20	30	546.5	<0.001
	Allergic conjunctivitis vs control	28	30	515.0	0.422

Pairwise comparisons were performed using two-sided Mann–Whitney U tests.
P-values were adjusted for three pairwise comparisons within each eyelid using the Bonferroni method.
BKC: blepharo-keratoconjunctivitis, RUL: right upper lid, RLL: right lower lid, LUL: left upper lid, LLL: left lower lid

*n= number of patients

Supplementary Table S1. Bonferroni-adjusted post hoc pairwise comparisons of meibomian gland loss among the BKC, allergic conjunctivitis, and control groups.
