# Supplementary material for: Comparison of Meibomian Gland Loss Between Pediatric Patients with Blepharo-Keratoconjunctivitis and Age-Matched Controls Using Non-Contact Meibography
Source: J Clin Med. 2026 Sep 6;15(17):6891. doi: 10.3390/jcm15176891 (PMC13566522; doi:10.3390/jcm15176891)
Supplement: Supplementary file 1 [file jcm-15-06891-s001.zip › Supplementary Table S2_multivariable regression.pdf]

Outcome	BKC	Allergic conjunctivitis	Age (per year)	Female sex	n	R^2	Adjusted R^2	Model p-value
RUL MG loss	33.57 (4.53), (24.54, 42.60)	0.37 (3.98), (−7.56, 8.30)	−0.14 (0.52), (−1.18, 0.90)	1.62 (3.67), (−5.69, 8.93)	75	0.515	0.487	<0.001
p-value	<0.001	0.926	0.791	0.660				
RLL MG loss	23.11 (4.42), (14.29, 31.92)	6.14 (3.95), (−1.74, 14.01)	−0.33 (0.51), (−1.35, 0.70)	−4.15 (3.58), (−11.29, 2.98)	81	0.268	0.230	<0.001
p-value	<0.001	0.125	0.528	0.250				
LUL MG loss	38.01 (4.90), (28.24, 47.78)	1.01 (4.14), (−7.25, 9.28)	0.14 (0.59), (−1.03, 1.32)	3.76 (3.81), (−3.85, 11.37)	76	0.550	0.524	<0.001
p-value	<0.001	0.808	0.811	0.328				
LLL MG loss	33.46 (5.12), (23.26, 43.67)	4.46 (4.64), (−4.77, 13.70)	−0.11 (0.59), (−1.29, 1.06)	1.69 (4.17), (−6.61, 10.00)	78	0.421	0.389	<0.001
p-value	<0.001	0.339	0.846	0.685				

Separate multivariable linear regression models were fitted for each eyelid.

BKC: blepharo-keratoconjunctivitis, RUL: right upper lid, RLL: right lower lid, LUL: left upper lid, LLL: left lower lid

*n= number of patients

Supplementary Table S2. Multivariable linear regression analyses of meibomian gland loss in each eyelid. Diagnostic group, age, and sex were included as covariates. The control group and male sex were used as the reference categories. Values are adjusted β coefficients (standard errors), followed by 95% confidence intervals in parentheses: β (SE), (lower 95% CI, upper 95% CI).
